# Identification of unobservable behavior in stochastic discrete event systems with a low number of sensors

**DOI:** 10.1016/j.mex.2023.102316

**Published:** 2023-08-07

**Authors:** Rubén Santillán-Mosquera, Mariela Muñoz-Añasco

**Affiliations:** Facultad de Ingeniería Electrónica y Telecomunicaciones, Grupo de Automática, Universidad Del Cauca, Popayán, Cauca, Colombia

**Keywords:** Modeling, Interpreted petri net, Stochastic discrete event system, Identification of unobservable behavior, Petri net language, Method to identify unobservable behavior in stochastic discrete event systems with a low number of sensors.

## Abstract

Dynamic discrete event systems (DDES) are systems that evolve from the asynchronous occurrence of discrete events. Their versatility has become a critical modeling tool in different applications. Finding models that define the behavior of DES is a topic that has been addressed from different approaches, depending on the type of system to be modeled and the model's objective. This article focuses on the identification of timed models for stochastic discrete event systems. The identified model includes both observable and unobservable behavior. The objective of the method is achieved through the following steps:

• Identifying the sequences of events observed at different time instances during the closed-loop operation of the system (observed language),
• Inferring the stochastic behavior of time between events and modeling the observable behavior as a stochastic timed Interpreted Petri Net (st-IPN),
• and finally, inferring the non-observable behavior using the language projection operation between the observed language and the language generated by the st-IPN.

This method has novel aspects because it uses timed events, can be applied to systems with a low number of sensors and can infer unobservable behavior for any sequence of events.

## Graphical abstract

**Figure:**
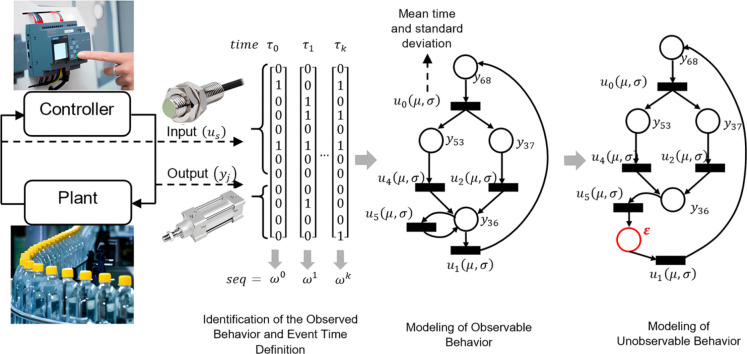

Specifications table

**Table:** 

Subject area:	Engineering
More specific subject area:	Discrete event system modeling
Name of your method:	Method to identify unobservable behavior in stochastic discrete event systems with a low number of sensors.
Name and reference of original method:	D. M. Muñoz, A. Correcher, E. García, and F. Morant, “Identification of stochastic timed discrete event systems with st-IPN,” Math. Probl. Eng., vol. 2014, 2014. 10.1155/2014/835312
Resource availability:	N.A.

## Introduction

A DES model can be developed from an expert's knowledge of the system by applying refinement techniques (top-down design) or modular composition (bottom-up design), and identification methods. The identification methods start from the observed input-output signals of the system, which generate sequences of events that define the system's behavior [1]. The set of sequences forms the language of the system [2]. Additionally, when the events' time is considered, this is called timed language. Identification algorithms have the observed language as the input and a model representing the observed language as the output [3,4]; in turn, timed identification algorithms further infer the stochastic behavior of the transitions.

The observed language can be found by measuring the changes in the actuator signals, such as from switches and valves, or sensor signals, which change according to specific actions. The representation of the observed language as a DES is applied in the modeling of industrial processes, production systems, robotics, manufacturing processes, traffic systems, biological and chemical processes [5], and telecommunication networks [6], among others. When these processes have a large number of sensors and actuators, the observable behavior is generally sufficient to produce a model close to the real behavior of the system; however, when the system has a shortage of these reading and control elements, the language cannot be used to record the changes that occur during the process efficiently. This issue gives rise to unobservable behavior [7] that is not easily identifiable.

The identification of the unobservable behavior of DES represented as PNs is a problem that has been studied to a limited extent. This unobservable behavior may be due to unobservable states or unobservable transitions [8], [9], [10] in a closed-loop DES. The focus of this paper is the identification of unobservable states. In the literature reported on this approach, the proposed methods identify observable behavior as partial Petri nets and the model is completed by adding unobservable places. The addition of unobservable behavior is performed from the analysis of the observable IPN structure or combined with knowledge models, i.e., generated by experts, [10,11], for example, in [11], the model identified from the signal sequences is modeled as a Signal Interpreted Petri net (SIPN), then from the sequence represented in the SIPN an argued reachability graph is constructed and based on that graph, In [12] proposes a method for discovering sequential and concurrent relationships between events in the observed sequence, when two transitions are observed consecutively and one is systematically preceded by the other there is a sequential relationship and when two transitions have been observed consecutively in both orders there is a concurrent relationship, the unobservable places represent the internal behavior of the system and are added so that the final model represents the sequence of events, in the work [13], the identified events that do not match the sequence of events can be considered unobservable events and are added to the model identified from the generation of T-invariants at the end, in [14] the observable behavior of a closed-loop, plant-controller system is modeled from the exchange of physical signals between the plant and the controller, the expiration of a timer, are assumed to be internal events whose occurrence generates the unobservable behavior, observable behavior is identified as IPN fragments and unobservable places are added for the model to represent the observed event sequences. Another approach is based on language theory, [15], they discover unobservable behavior based on the projection of the firing sequence obtained in the first step on subalphabets, and finds specific patterns that are characteristic of the dependency relationships between the transition firings. Unlike the previous approaches, in [7,16], once the sequence of events has been observed, they first perform a language reduction from a synthesis approach against a design parameter of word size r, then, they define a language superior to that of the identified net, from the observed marking achieved during the firing of the sequence of transitions, finally an optimization approach based on integer linear programming is used to find the unobservable behavior.

In general, these approaches propose inferring unobservable behavior by discovering dependence relationships in the observed sequences, comparing them with knowledge models, or applying optimization techniques. Still, they do not report specific instrumentation conditions of the automatic system to be identified. In the present work, we propose to infer the unobservable behavior without using the observable IPN structure; moreover, the main novelty lies in the fact that the proposed method can be applied to a reactive DES, that is, to a closed-loop system consisting of a plant and a controller exchanging signals, where the plant has a low number of sensors; and, to identify the observable and unobservable behavior, the input information is not only constituted by the sequences of the changes in the I/O signals but also the time in which these changes occur is taken into account. Based on these characteristics, the method can be applied in different industrial automation systems, such as communication systems, transport systems, systems where timing information becomes more critical, or production systems where sensorization levels are low.

The article is organized as follows: section 2 presents the conceptual basis of the proposal, section 3 describes the method for identifying unobservable behavior, section 5 presents the method validation and discussion, and finally, section 6 presents the conclusions of the work.

## Basic concepts

Modeling the behavior of a discrete event system (DES) is essential for conducting functional analysis studies, carrying out performance evaluations, and applying simulation techniques, among other applications. The dynamics of these systems are represented based on the asynchronous occurrence of discrete events; some of these events are controlled, whereas others are not, and some of these events are observed by sensors, whereas others are not [2]. A reactive DES is a closed-loop system consisting of a plant and a controller exchanging signals. The most commonly used representation formalisms for DESs are Automata and Petri nets (PNs). PNs offer advantages for modeling DES because they represent concurrent, asynchronous, distributed, parallel, nondeterministic and/or stochastic behavior. This work is based specifically in two types of PNs are Interpreted Petri nets (IPN) and timed Petri nets (t-PN) [17].

The following are the basic concepts and basic notations used in the article.

Definition 1 *Petri Nets (PN)*.

PN are models capable of describing the total information flow of a system with concurrent and distributed processes, and they are widely used to model DESs. PNs provide compact models and capture important characteristics of DESs, such as concurrency, synchronism, causal relationships, and shared resources.

The PN structure N is a bipartite digraph represented by the five-tuple $N=(P,TR,Pre,Post,\;M_{0})$, where P is a set of places with cardinality np, TR is a set of transitions with cardinality ntr, and Pre:P×TR→Z, Post:TR×P→Z are the *Pre*- and Post-incidence matrices, respectively, which specify the arcs connecting the places and transitions. Matrix C=Post−Pre is the np×ntr incidence matrix of the net. The marking function M:P→Z represents the number of tokens residing in each place, and $M_{0}$ is the initial marking [18].

### Background

The present work is based on the modeling proposal presented by [1]; some of the considerations of this proposal are the following:

Definition 2 Closed loop system

A closed-loop system between plant and controller is considered; the inputs to the plant are the control commands and the outputs are the sensor readings. Both inputs (u) and outputs (y) are represented as vectors with binary values. Each input and output have a particular value at each instant. For example, given the of control commands $Cc=\{cc_{1},cc_{2}\}$ sensors $Sr=\{s_{1},s_{2}\}$. If the readings observed at an instant of time, $\tau_{i}$ are $cc_{1}=0$, $cc_{2}=0$, $s_{1}=1$ and $s_{2}=0$, then u=[00] and y=[10].

Definition 3 Input – Output (I/O) Vector

For simplicity in representation, I/O vectors are encoded using their decimal representation; therefore, an input (output) symbol is defined as the set of control command values (sensor readings) at an instant of time in decimal representation. Therefore, an I/O symbol is defined as:

(1) $$\left(u_{s},y_{j}\right)$$

where, s stands for the input symbol in decimal, and $s=0;\cdots ,2^{m}-1$, with $u_{0}=\left[\vec{0}\right],\cdots ,u_{2^{m}-1}=\left[\vec{1}\right]$; the same applies to $y_{j}$, j stands for the output symbol in decimal, and $j=0;\cdots ,2^{n}-1$, with $y_{0}=\left[\vec{0}\right],\cdots ,u_{2^{n}-1}=\left[\vec{1}\right]$; *m* and *n* are the numbers of inputs and outputs, respectively.

Definition 4 Interpreted Petri Net (IPN)

An IPN is a tuple Q=(N,U,Y,λ,φ), where N is a PN, $U=\{u_{0},\cdots ,u_{2^{m}-1}\}$ is the input alphabet, $u_{s}$ is an input symbol, and m is the number of inputs; $Y=\{y_{0},\cdots ,y_{2^{n}-1}\}$ is the output alphabet, $y_{j}$ is an output symbol, and n is the number of outputs; λ:TR→U is a labeling transition function that assigns an input symbol to each transition and φ:P→Y is an output function that assigns an output symbol to each place.

The system alphabet Ω relates the I/O symbols; specifically, Ω=U·Y. Therefore, an ω event such that ω∈Ω, is on the form $\omega =u_{s}y_{j}$, according to Eq. (1).

Definition 5 Event

A new event, ω, is generated when there is a change in $u_{s}$, $y_{j}$, or both. For the above example, If the readings observed at different time instants $(\tau_{i})\;$are



Then, the initial event is $\omega^{0}=\left(\left[00],[00\right]\right)$, i.e. $\omega^{0}=\left(u_{0}y_{0}\right)$; and the following events are represented as follows: There is an event from $\tau_{0}$ to $\tau_{1}$ because there is a change in both the input and output signals, $\omega^{1}=\left(u_{2}y_{2}\right)$; there is another event from $\tau_{1}$ to $\tau_{2}$ because there is a change in the input signal but not in the output signal, $\omega^{2}=\left(u_{3}y_{2}\right)$, there is another event from $\tau_{2}$ to $\tau_{3}$ because there is a change in the output signal but not in the input, $\omega^{3}=\left(u_{3}y_{3}\right)$ and there is another event from $\tau_{3}$ to $\tau_{4}$ because the input signals change and a change in an output signal is generated, $\omega^{4}=\left(u_{0}y_{1}\right)$.

Definition 6 Time event

A timed event is defined as

(2) $$\omega^{i}=\left(u_{s}y_{j}\right)t_{i}$$

where $t_{i}=\left|\tau_{i}-\tau_{i-1}\right|$, and $\tau_{i}$ is the time instant when the event *i* occurred. When the DES is stochastic, the timed information is represented by the mean and standard deviation calculated from different operating cycles of the system.

(3) $$\omega^{i}=\left(u_{s}y_{j}\right)\left(\mu_{i},\sigma_{i}\right)$$

A sequence is a concatenation of timed events organized in a timeline:

(4) $$seq_{q}^{obs}=\omega^{0}\cdots \omega^{k}=\left({\left(u_{s}y_{j}\right)}^{0}\left(\mu_{0},\sigma_{0}\right)\right)\cdots \left({\left(u_{s}y_{j}\right)}^{k}\left(\mu_{k},\sigma_{k}\right)\right)$$

where q:1⋯nq, nq is the number of observed sequences.

The set of sequences constitutes the observed behavior of the system or the observed language, $L_{o}$.

(5) $$L_{o}=\left\{seq_{1}^{obs}\cdots seq_{nq}^{obs}\right\}$$

Definition 7 Stochastic Timed Interpreted Petri net (st-IPN)

A st-IPN is a structure represented by stQ=(Q,Ω,δ), where Q=(N,U,Y,λ,φ) is an IPN, N,U,Y have the same meaning as Definition 4, but the input function λ is defined as λ:TR→U.δ, a labeling function that assigns an input symbol and a density function to each transition, and φ is defined as φ:P→Y, where φ is isomorphic over $y_{j}$.

The statistical behavior of the transitions, δ, is inferred from the time data observed during many cycles of operation of the system.

Definition 8 *Language generated by a st-IPN* $\left(L_{g}\right)$

$L_{g}$ is composed of all event sequences $(seq_{q}^{g})\;$that evolve the st-IPN. That is

(6) $$L_{g}=\left\{seq_{1}^{g}\cdots seq_{nq}^{g}\right\}$$

where $seq_{q}^{g}=\omega^{0}\cdots \omega^{k}$ and $\omega^{k}$ is an event as defined in the Eq. (3).

Definition 9 *Input language* (L(in))

The L(in) constitutes the sequences of the input symbols of a language. According to [3], the L(in) is found from the language projection operation as follows:

(7) $$P_{U}:L\to L\left(in\right)$$

where, $P_{U}\left(\lambda \left(tr_{K}\right)\varphi \left(p_{k}\right)\right)=\lambda \left(tr_{K}\right)\;if\;\lambda \left(tr_{K}\right)\in U$ and $P_{U}\left(\lambda \left(tr_{K}\right)\varphi \left(p_{k}\right)\right)=\varepsilon \;if\;\lambda \left(tr_{K}\right)\notin U$.

Definition 10 *Output language* (L(out))

The L(out) constitutes the sequences of the output symbols of the language. According to [3], the L(out) is found from the language projection operation as follows:

(8) $$P_{Y}:L\to L\left(out\right)$$

where $P_{Y}\left(\lambda \left(tr_{K}\right)\varphi \left(p_{k}\right)\right)=\varphi \left(p_{k}\right)\;if\;\varphi \left(p_{k}\right)\in Y$ y $P_{Y}\left(\lambda \left(tr_{K}\right)\varphi \left(p_{k}\right)\right)=\varepsilon \;if\;\varphi \left(p_{k}\right)\notin Y$.

### Problem statement

One way to find the behavioral model of a DES is from identification methods. The identified models have an observable and an unobservable behavior. The observable behavior is constructed from the system's input and output (I/O) signals. The unobservable behavior is inferred from the I/O signals; this behavior is generated when there are changes in the internal dynamics of the system, without having generated changes in the I/O signals; one cause is that the system is not entirely sensorized, an aspect that becomes important in industrial applications. The models obtained through identification have different purposes, such as fault diagnosis, validation and synthesis of controllers, so models that represent the system's dynamics as real as possible are required. Proposals to identify unobservable behavior under the Petri net formalism have been reported in the literature from different approaches: one approach compares the observable behavior with previous models given by experts and adds the missing sites, another approach conditions the observable NP to the fulfillment of T-Invariants; and another approach is the one presented in [15] where the unobservable part is determined by projecting the activation sequences of the sub-alphabets to discover specific patterns, which are characteristic of the dependency relationships between transitions, an approach that has a weak point when in the sequence of events no patterns can be found, for example, strings of events that only occur once in the system and also, in very long sequence strings that proposal presents a problem in the choice of the parameter of the size of the languages.

Considering the above, this paper proposes a method to infer the unobservable behavior for stochastic DES with a low number of sensors, from timed event sequences, observed during many cycles of system operation and that allows modeling event sequences independent of the number of repetitions it has, based on the proposal to identify the observable behavior presented in [1].

## Method details

The proposed method consists of two stages: the identification of the structure of an st-IPN (see Definition 7) from sequences of timed events observed as defined in Definition 6, of a system as described in Definition 2, and then the addition of the unobservable behavior to the structure of the identified st-IPN based on the projection of languages.

Unlike reference [1], In the present proposal the system is not divided into subsystems; furthermore, the identification algorithm works offline.

### Identification of the observed behavior

(1) To observe the operation of the system to be identified for several cycles and to record the information of the input/output signals, as well as the time, in a table as shown in Table 1.

**Table 1:** I/O readings. Algorithm for identification of the observable.

$\tau_{i}$	control commands				sensor readings			
	$cc_{1}$	$cc_{2}$	⋯	$cc_{m}$	$sr_{1}$	$sr_{2}$	⋯	$sr_{n}$
0	{0,1}	{0,1}		{0,1}	{0,1}	{0,1}		{0,1}
⋮
(2) Generate the initial event: The generation of the initial event $\omega^{0}=\left(u_{s}y_{j}\right)\tau_{0}$, $u_{s},y_{j}$ is the reading of the input and output signals at instant 0, respectively,according to Eq. (1).
(3) Event generation: A new event is generated when at least one of the signals has changed, such as when $\omega^{i}\neq \omega^{i-1}$, meaning. The time of the event is $t_{i}=\tau_{i}-\tau_{i-1}$.
(4) Identify the observed sequences of events based on the requirements of Eq. (4).
(5) Define the observed language $\left(L_{o}\right)$ as the set of observed sequences.

To demonstrate the method, an introductory example is presented. Let there be a closed-loop system with eight signals emitted by the controller and a plant with only four sensors. The initial event is $\omega^{0}=\left(u_{68}y_{0}\right)\tau_{0}$, that is $u_{68}=\left[,010,001,00\right]$, $y_{0}=\left[0000\right]$, $\tau_{0}=0$. The system is observed for some time. The first change of the I/O signals is identified at 11-time units, in which $u_{68}$ becomes $u_{196}$, thus generating an $\omega^{1}=\left(u_{196}y_{0}\right)11$ event., $\omega^{1}=\left(u_{196}y_{0}\right)11$, All detected changes constitute the sequence of events $seq_{1}$, where

$${\text{seq}}_{1}^{obs}=\left(\left(u_{34}y_{0}\right)0\right)\left(\left(u_{196}y_{0}\right)11\right)\left(\left(u_{192}y_{0}\right)5\right)\left(\left(u_{193}y_{1}\right)8\right)\left(\left(u_{33}y_{3}\right)1\right)\left(\left(u_{35}y_{12}\right)15\right)\left(\left(u_{34}y_{0}\right)10\right).$$

#### Event time definition

The times of the events in each sequence are stored in a matrix, where the number of columns is the number of cycles observed in the sequence, and each row represents the observed time of the event. Once the system has been observed for several cycles, the mean and deviation of the time in each event and in each sequence can be determined.

For the introductory example, the observed timing behavior is as follows:

$$L_{o}=\left(\left(u_{68}y_{0}\right)\left(1,0\right)\right)\left(\left(u_{196}y_{0}\right)\left(8.3,0.2\right)\right)\left(\left(u_{192}y_{0}\right)\left(5.6,0.3\right)\right)\left(\left(u_{193}y_{1}\right)\left(9.2,0.25\right)\right)\left(\left(u_{33}y_{3}\right)\left(1,0\right)\right)\left(\left(u_{35}y_{12}\right)\left(12.2,0.06\right)\right)\left(\left(u_{34}y_{0}\right)\left(9.95,0.2\right)\right),$$

that is $L_{o}=\left\{\left(\omega^{0}\omega^{1}\omega^{2}\omega^{3}\omega^{4}\omega^{5}\omega^{6}\right)\right\}$.

### Modeling of observable behavior

**Fig. 1:**
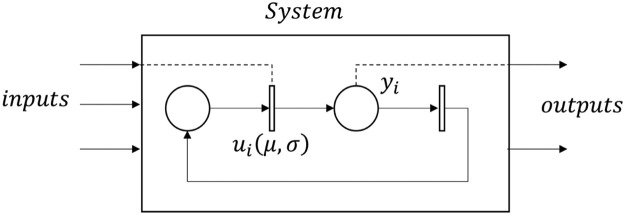
Representation of an st-IPN.

Each of the identified sequences is represented in an st-IPN. The input signals, $u_{s}$, are associated as the labels of the transitions, as well as the mean and standard deviation, and the output signals, $y_{j}$, are the labels of the places (Fig. 1).

The identification of the observable st-IPN is made based on Algorithm 1.

#### Elimination of transition self-loops

**Fig. 2:**
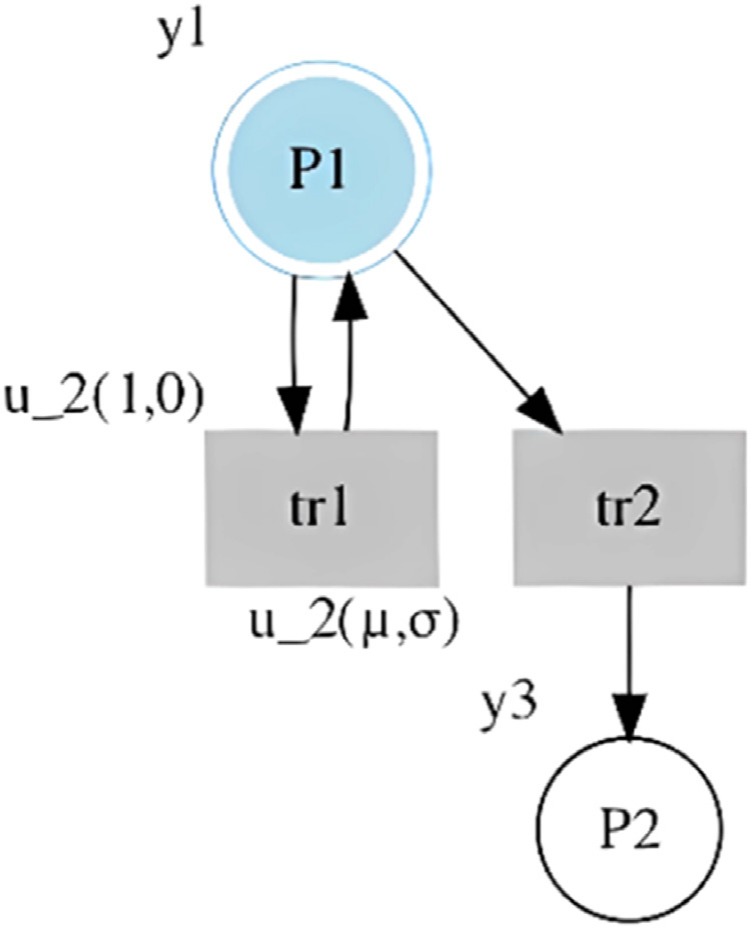
Example of self-loop elimination.

Once the st-IPN is available, a net reduction process is performed based on the elimination of the transition self-loops. Specifically, a transition tr is eliminated if its places pre and post are the same (·tr=tr·). Additionally, a transition is eliminated if the associated time is deterministic, and if there is another transition with the same input signals but with a different output place. Indeed, these transitions are assumed to be transitions associated with an event that has been generated due to the settling time in the response of the sensors. Fig. 2 presents the structure of an st-IPN in which the $tr_{1}$ is eliminated.

### Modeling of unobservable behavior

The present proposal for modeling unobservable behavior is based on analyzing the generated language and the input and output languages of the st-IPN obtained in the previous step.

The following procedure is proposed to model unobservable behavior.

(1) Find the language generated, ($L_{g}),$ by the identified st-IPN defined as in Eq. (6).
(2) Find the input and output languages of the observed and st-IPN-generated languages. The input and output languages of the observed and st-IPN generated languages are found based on Eqs. (7) and 8. Therefore, the input language will be $L{\left(in\right)}_{seq_{g}}=\left(u_{s}^{0}\left(\mu_{0},\sigma_{0}\right)\cdots u_{s}^{k}\left(\mu_{k},\sigma_{k}\right)\right)$ and the output language will be $L{\left(out\right)}_{seq_{g}}=\left(y_{j}^{0}\cdots y_{j}^{k}\right)$.
(3) Identify event subsequences where unobservable places may be present. A sub-sequence of timed events, such that the L(out) is composed of identical output symbols and in its L(in), the input symbols are not identical, it may be that there are unobservable places between the transitions involved in the sub-sequence. This phenomenon occurs because it is not possible to model the states to which the system has evolved from the input signals due to the low number of sensors.
(4) Infer unobservable places from observable behavior. It is proposed to infer the unobservable places between two transitions if the sub-sequence identified in previous step two or more consecutive transitions with different input symbols are generated starting from the same place pre.
(5) Add the unobservable places to the previously identified st-IPN.

Applying the above procedure to the introductory example, the observed language is the following:

$$L_{o}=\{\left(\left(u_{34}y_{0}\right)\left(1,0\right)\right)\left(\left(u_{196}y_{0}\right)\left(8.3,0.2\right)\right)\left(\left(u_{192}y_{0}\right)\left(5.6,0.3\right)\right)\left(\left(u_{193}y_{1}\right)\left(9.2,0.25\right)\right)\left(\left(u_{33}y_{3}\right)\left(1,0\right)\right)\left(\left(u_{35}y_{12}\right)\left(12.2,0.06\right)\right)\left(\left(u_{34}y_{0}\right)\left(9.95,0.2\right)\right)\}.$$

$$L_{o}=\left\{\left(\omega^{0}\omega^{1}\omega^{2}\omega^{3}\omega^{4}\omega^{5}\omega^{6}\right)\right\}.$$

**Fig. 3:**
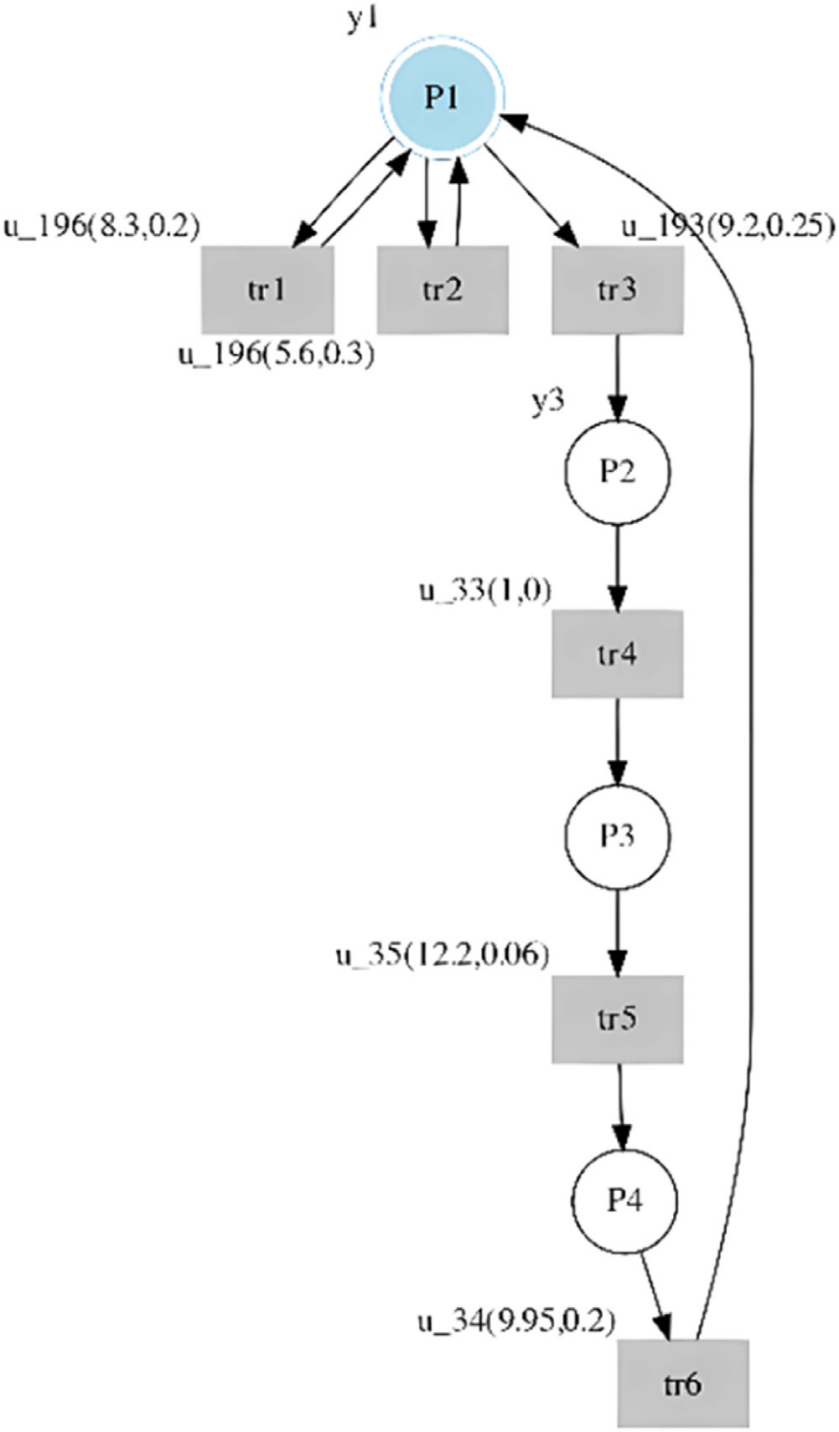
St-IPN identified for the introductory example language.

And applying Algorithm 1 to this timed sequence, the st-IPN identified is shown in Fig. 3.

The language generated by the st-IPN is composed of the following sequences: $L_{g}=\left\{seq_{1},\;seq_{2},seq_{3}\right\}$; where $seq_{1}=\left(\omega^{0}\omega^{1}\omega^{2}\omega^{3}\omega^{4}\omega^{5}\omega^{6}\right)$; $seq_{2}=\left(\omega^{2}\omega^{3}\omega^{4}\omega^{5}\omega^{6}\right)$; and $seq_{3}=\left({\left\{\omega^{0},\omega^{1}\right\}}^{*}\omega^{2}\omega^{3}\omega^{4}\omega^{5}\omega^{6}\right)$, (${\left\{\omega^{0},\omega^{1}\right\}}^{*}$ is the Klein closure).

As can be seen, the language generated by the st-IPN represents behaviors that are not identified in the system. Since the observed language of a DES defines its behavior, it is a primary condition that the models generate the same language. When a DES is not sufficiently sensorized, it is common to be faced with the problem of identifying models that do not generate the same language, but including unobservable places can solve this issue. For this purpose, the observed and generated L(in) and L(out) languages are analyzed to establish between which transitions the unobservable places should be included.

For introductory example, the input and output languages observed are as follows:

$$L{\left(out\right)}_{o}=\left\{\left(y_{o}y_{o}y_{o}y_{1}y_{3}y_{12}y_{0}\right)\right\}.$$

$$\;L{\left(in\right)}_{o}=\{\left(u_{34}\left(1,0\right)\right)\left(u_{196}\left(8.3,0.2\right)\right)\left(u_{192}\left(5.6,0.3\right)\right)\left(u_{193}\left(9.2,0.25\right)\right)\left(u_{33}\left(1,0\right)\right)\;\left(u_{35}\left(12.2,0.06\right)\right)\left(u_{34}\left(9.95,0.2\right)\right)\}.$$

And, the input and output languages generated by st-IPN are as follows:

$$L{\left(out\right)}_{g}=\{\left(y_{o}y_{o}y_{o}y_{1}y_{3}y_{12}y_{0}\right),\left(y_{o}y_{1}y_{3}y_{12}y_{0}\right),({\left\{y_{o},y_{o}\right\}}^{*}y_{o}y_{1}y_{3}y_{12}y_{0})\}.$$

$$\begin{array}{ccc} L{\left(in\right)}_{o} & = & \{\left(\left(u_{34}\left(1,0\right)\right)\left(u_{196}\left(8.3,0.2\right)\right)\left(u_{192}\left(5.6,0.3\right)\right)\left(u_{193}\left(9.2,0.25\right)\right)\left(u_{33}\left(1,0\right)\right)\left(u_{35}\left(12.2,0.06\right)\right)\left(u_{34}\left(9.95,0.2\right)\right)\right),\\ & & \left(\left(u_{192}\left(5.6,0.3\right)\right)\left(u_{193}\left(9.2,0.25\right)\right)\left(u_{33}\left(1,0\right)\right)\left(u_{35}\left(12.2,0.06\right)\right)\left(u_{34}\left(9.95,0.2\right)\right)\right),\\ & & \left({\left\{\left(u_{34}\left(1,0\right)\right),\left(u_{196}\left(8.3,0.2\right)\right)\right\}}^{*}\left(u_{192}\left(5.6,0.3\right)\right)\left(u_{193}\left(9.2,0.25\right)\right)\left(u_{33}\left(1,0\right)\right)\left(u_{35}\left(12.2,0.06\right)\right)\left(u_{34}\left(9.95,0.2\right)\right)\right)\} \end{array}$$

**Fig. 4:**
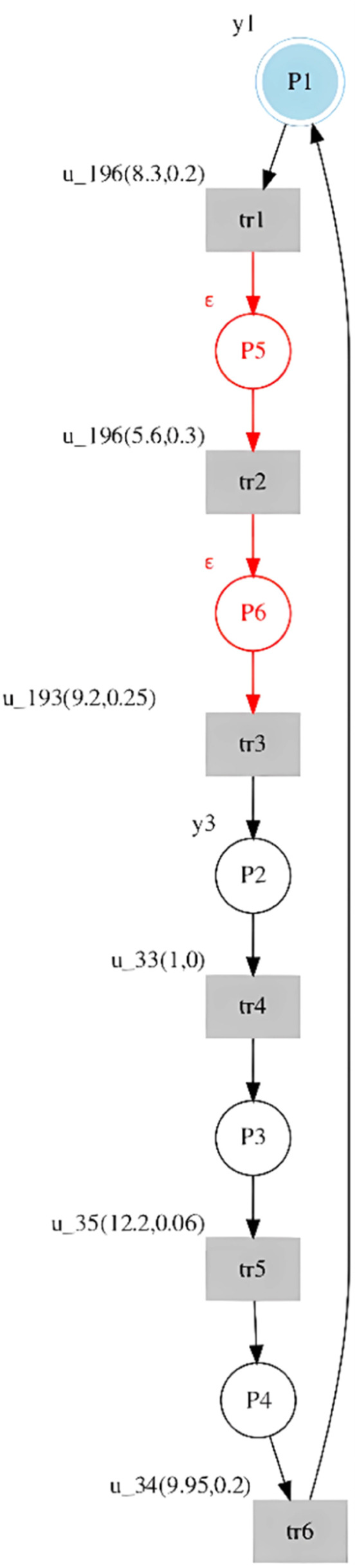
St-IPN with observable and unobservable places from the introductory example.

By analyzing the generated input and output languages, it can be verified that the $seq_{1}$ observed and $seq_{1}$ generated by the st-IPN are the same. Furthermore, since the observed $seq_{1}$ models all the system's behavior, the generated $seq_{2}$ and $seq_{3}$ sequences are not part of the system behavior. In addition, each input of the sub-sequence $\left(u_{34}\left(1,0\right)\right)\left(u_{196}\left(8.3,0.2\right)\right)\left(u_{192}\left(5.6,0.3\right)\right)$ of the $seq_{1}$ generated has the same output, $y_{o}$. Therefore, the procedure proposed in this paper can be applied. An unobservable place was inferred between $tr_{1}$ and $tr_{2}$ and another between $tr_{2}$ and $tr_{3}$. The st-IPN with both observable and unobservable behavior is shown in Fig. 4, with the added behavior specified in red.

### Contribution

The representation formalism used to model behavior based on an st-IPN, which allows modeling changes in output signals that depend on changes in input signals, assigning labels to transitions and places; but also capturing the stochastic behavior of events. This feature has advantages over proposals that model only the behavior of signals through IPNs [15,19] or only the timed behavior [3].

In addition, the inference of unobservable behavior based on the analysis of the timed sequences of input events allows the addition of places whose dynamics is related to the evolution of the internal state of the plant caused by low plant sensing or by timed responses, without incurring computational costs that are generated when optimization techniques or invariant calculations are applied.

## Method validation

This section presents the proposed method's results in a case study. The case study is an industrial application with behavior that evolves from states and whose behavioral model is required as a DES. Its objective is to glue a soft pad over an armrest. The line has a conveyor, a glue injector with a sliding movement, a pad chute with an inclined belt, a pad dispenser and placer, a pad press station whit a vibrator, cylinders along the line that perform the function of barrier and traffic control, an automatic wrapper station, and an HMI screen where the parameterization, control, and supervision of the entire system is performed. The plant under study has a stochastic behavior and is instrumented with six sensors and 16 actuators which are listed to following:

**Sensors**: Reflex Sensor 1, Reflex Sensor 2, Reflex Sensor 3, Reflex Sensor 4, Proximity Sensor 1 and Proximity Sensor 2.
**Actuators**: Barrier Cylinder 1, Barrier Cylinder 2, Run Glue Injector, Glue spread Cylinder, Barrier Cylinder 3, Barrier Cylinder 4, Positioner Cylinder 1, Pad Placer cylinder, Barrier Cylinder 5, Press Cylinder, Positioner Cylinder 2, Run vibration and Run spinner wrapper.

It is desired to find the model that represents the observable and unobservable behavior as a DES. For this purpose, it is proposed to use the method presented in this article.

When observing the operation of the plant from the initial event $\left(u_{9},y_{0}\right)$, it was found that its behavior is defined by three sequences of events, which are shown in Table 2.

**Table 2:** Behavioral sequences of the case study.

$s_{1}$ sequence		$s_{2}$ sequence		$s_{3}$ sequence	
I/O symbol	(μ,σ)	I/O symbol	(μ,σ)	I/O symbol	(μ,σ)
$\left(u_{6},y_{0}\right)$					
$\left(u_{30},y_{2}\right)$	(18.1,6.3)	$\left(u_{30},y_{2}\right)$	(18.1,6.3)	$\left(u_{30},y_{2}\right)$	(18.1,6.3)
$\left(u_{126},y_{2}\right)$	(4,0)	$\left(u_{126},y_{2}\right)$	(4,0)	$\left(u_{126},y_{2}\right)$	(4,0)
$\left(u_{30},y_{2}\right)$	(1,0)	$\left(u_{30},y_{2}\right)$	(1,0)	$\left(u_{30},y_{2}\right)$	(1,0)
$\left(u_{14},y_{2}\right)$	(9.9,2.8)	$\left(u_{14},y_{2}\right)$	(9.9,2.8)	$\left(u_{14},y_{2}\right)$	(9.9,2.8)
$\left(u_{14},y_{0}\right)$	(1,0)	$\left(u_{14},y_{0}\right)$	(1,0)	$\left(u_{14},y_{0}\right)$	(1,0)
$\left(u_{14},y_{4}\right)$	(1,0)	$\left(u_{14},y_{4}\right)$	(1,0)	$\left(u_{14},y_{4}\right)$	(1,0)
$\left(u_{22},y_{0}\right)$	(1.1,0.3)	$\left(u_{22},y_{0}\right)$	(1.1,0.3)	$\left(u_{22},y_{0}\right)$	(1.1,0.3)
$\left(u_{278},y_{8}\right)$	(4.6,0.5)	$\left(u_{278},y_{8}\right)$	(4.6,0.5)	$\left(u_{278},y_{8}\right)$	(4.6,0.5)
$\left(u_{278},y_{0}\right)$	(1,0)	$\left(u_{278},y_{0}\right)$	(1,0)	$\left(u_{278},y_{0}\right)$	(1,0)
$\left(u_{406},y_{0}\right)$	(2.2,0.4)	$\left(u_{406},y_{0}\right)$	(2.2,0.4)	$\left(u_{406},y_{0}\right)$	(2.2,0.4)
$\left(u_{918},y_{0}\right)$	(4.2,0.4)	$\left(u_{918},y_{0}\right)$	(4.2,0.4)	$\left(u_{918},y_{0}\right)$	(4.2,0.4)
$\left(u_{22},y_{0}\right)$	(3.3,0.5)	$\left(u_{22},y_{0}\right)$	(3.3,0.5)	$\left(u_{22},y_{0}\right)$	(3.3,0.5)
$\left(,u_{,164,06},y_{16}\right)$	(6.3,0.5)	$\left(,u_{,164,06},y_{16}\right)$	(6.3,0.5)	$\left(,u_{,164,06},y_{16}\right)$	(6.3,0.5)
$\left(,u_{,164,06},y_{0}\right)$	(1,0)	$\left(,u_{,164,06},y_{0}\right)$	(1,0)	$\left(,u_{1,640,66},y_{0}\right)$	(1,0)
$\left(,u_{,317,66},y_{0}\right)$	(2.3,0.5)	$\left(,u_{,317,66},y_{0}\right)$	(2.3,0.5)	$\left(,u_{,317,66},y_{0}\right)$	(2.3,0.5)
$\left(u_{22},y_{0}\right)$	(2,0)	$\left(u_{22},y_{0}\right)$	(2,0)	$\left(u_{22},y_{0}\right)$	(2,0)
$\left(u_{22},y_{32}\right)$	(18.1,6.3)	$\left(u_{22},y_{32}\right)$	(18.1,6.3)	$\left(u_{9},y_{0}\right)$	(1,0)
$\left(u_{22},y_{0}\right)$	(4,0)	$\left(u_{22},y_{0}\right)$	(4,0)		
$\left(,u_{,327,90},y_{1}\right)$	(1,0)	$\left(,u_{,327,90},y_{1}\right)$	(18.1,6.3)		
$\left(u_{22},y_{0}\right)$	(9.9,2.8)	$\left(u_{22},y_{1}\right)$	(1,0)		
		$\left(u_{22},y_{0}\right)$	(9.9,2.8)		

**Fig. 5:**
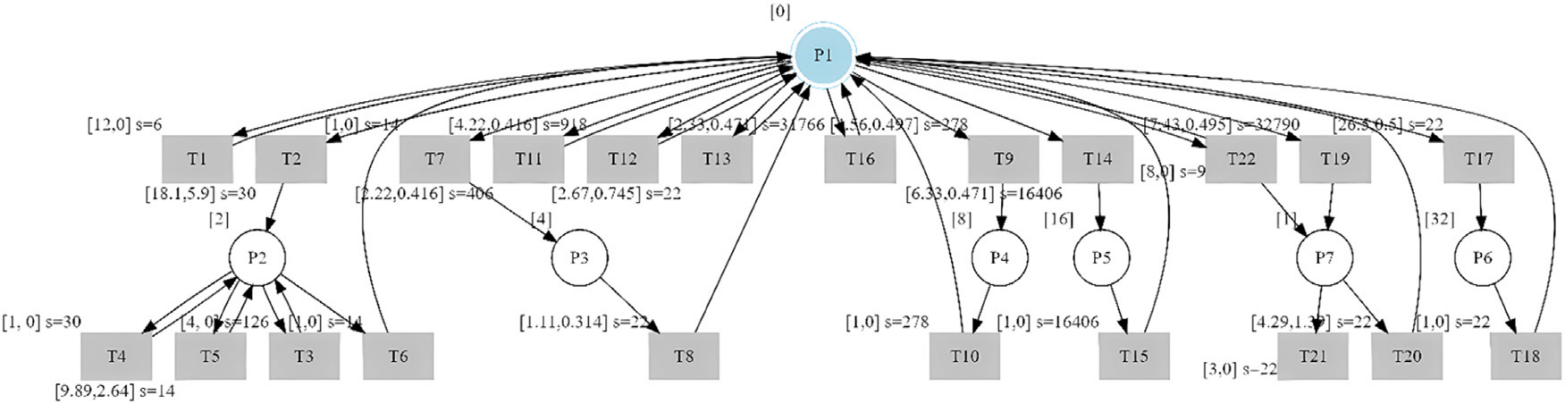
st-IPN of observable behavior of the case study.

Applying Algorithm 1 to the observed sequences, reported in Table 2, the resulting st-IPN is shown in Fig. 5, representing the observable behavior.

**Algorithm 1:** Algorithm for identification of the observable st-IPN.

$\mathrm{Input}{\;\mathrm{seq}}_{q}^{\mathrm{obs}}=\omega^{0}\omega^{1}\ldots \omega^{k};\omega^{i}=\left(u_{s}^{i},y_{j}^{i}\right)\left(\mu^{i},\sigma^{i}\;\right).$ OutputPre;Post;φ(P);λ(TR) $\mathrm{Initial}\;\mathrm{conditions}:\;\mathrm{np}=1;\;\mathrm{ntr}=0;\;\varphi \left(p_{1}\right)=y_{j}^{0};\;\mathrm{Pre}=\left[\right];\;\mathrm{Post}=\left[\right].$ fori=1:kk:numberofevents
$read\;\omega^{i}=\left(u_{s}^{i},y_{j}^{i}\right)\left(\mu^{i},\sigma^{i}\right)$ $if\;y_{j}^{i}\neq y_{j}^{i-1}\forall i\;then\;np=np+1;\left(new\;place\right)$
$\varphi \left(p_{np+1}\right)=y_{j}^{i};\;ntr=ntr+1;\;\lambda \left(tr_{ntr}\right)=u_{s}^{i}\left(\mu^{i},\sigma^{i}\right);$ $\overset{⇀}{pre}≔zeros\left(np+1,ntr\right):\;\overset{⇀}{pre}\left(np-1,ntr\right)=1;$ $update\;Pre\;with\;\overset{⇀}{pre};\;\overset{⇀}{post}≔zeros\left(np+1,ntr\right);\;\overset{⇀}{post}\left(np,ntr\right)=1;$ $update\;Post\;with\;\overset{⇀}{post};\;cp=np;\;ctr=ntr.$
$else\;if\;existing\;place;\;\overset{⇀}{pre}=Pre\left(:,ctr\right);\;ntr^{\prime }=ntr+1;$
$\lambda \left(tr_{ntr^{\prime }}\right)=u_{s}^{i}\left(\mu^{i},\sigma^{i}\right);$ forr=1:ntr
$if\;\lambda \left(tr_{r}\right)=\lambda \left(tr_{ntr^{\prime }}\right)\;and\;\overset{⇀}{pre}\left(cp,r\right)=1\;and\;\overset{⇀}{post}\left(cp,r\right)=1$
thenbreak(existingplace)
$else\;if\;\lambda \left(tr_{r}\right)\neq \lambda \left(tr_{ntr^{\prime }}\right)\wedge \overset{⇀}{pre}\left(cp,r\right)=1\wedge \overset{⇀}{post}\left(cp,r\right)=1$
$then\;ntr=ntr^{\prime }\;\left(new\;transition\right)$ $update\;Pre\;with\;\overset{⇀}{pre};\;\overset{⇀}{post}=Post\left(:,ntr\right);\;\overset{⇀}{post}\left(cp,ntr\right)=1;\;\lambda \left(tr_{ntr}\right)=\lambda \left(tr_{ntr^{\prime }}\right)$
endif
endfor
endif
endfor

For better visualization, the labels of the transitions are shown below.

$$\begin{array}{ccc} \lambda \left(tr_{1}\right) & = & u_{6}\left(12,0\right);\;\lambda \left(tr_{2}\right)=u_{30}\left(18.1,5.9\right).\;\;\lambda \left(tr_{3}\right)=u_{6}\left(12,0\right).\;\;\lambda \left(tr_{4}\right)=u_{30}\left(1,0\right).\;\;\lambda \left(tr_{5}\right)=u_{14}\left(9.9,2.64\right).\\ \lambda \left(tr_{6}\right) & = & u_{14}\left(1,0\right).\;\;\lambda \left(tr_{7}\right)=u_{14}\left(1,0\right).\;\;\lambda \left(tr_{8}\right)=u_{22}\left(1.1,0.3\right).\;\;\lambda \left(tr_{9}\right)=u_{278}\left(4.6,0.5\right).\;\;\lambda \left(tr_{10}\right)=u_{278}\left(1,0\right).\\ \lambda \left(tr_{11}\right) & = & u_{406}\left(2.2,0.4\right).\;\;\lambda \left(tr_{12}\right)=u_{918}\left(4.2,0.4\right).\;\;\lambda \left(tr_{13}\right)=u_{22}\left(2.7,0.7\right).\;\;\lambda \left(tr_{14}\right)=u_{16406}\left(6.3,0.5\right).\\ \lambda \left(tr_{15}\right) & = & u_{16406}\left(1,0\right).\;\;\lambda \left(tr_{16}\right)=u_{31766}\left(2.3,0.5\right).\;\;\lambda \left(tr_{17}\right)=u_{22}\left(26.3,0.5\right).\;\;\lambda \left(tr_{18}\right)=u_{22}\left(1,0\right).\\ \lambda \left(tr_{19}\right) & = & u_{32790}\left(7.4,0.5\right).\;\;\lambda \left(tr_{20}\right)=u_{22}\left(4.3,1.4\right).\;\;\lambda \left(tr_{21}\right)=u_{22}\left(8,0\right).\;\;\lambda \left(tr_{22}\right)=u_{22}\left(3,0\right).\;\;\lambda \left(tr_{23}\right)=u_{9}\left(8,0\right). \end{array}$$

The transition eliminated by transition self-loops due to settling time is $tr_{5}$.

**Fig. 6:**
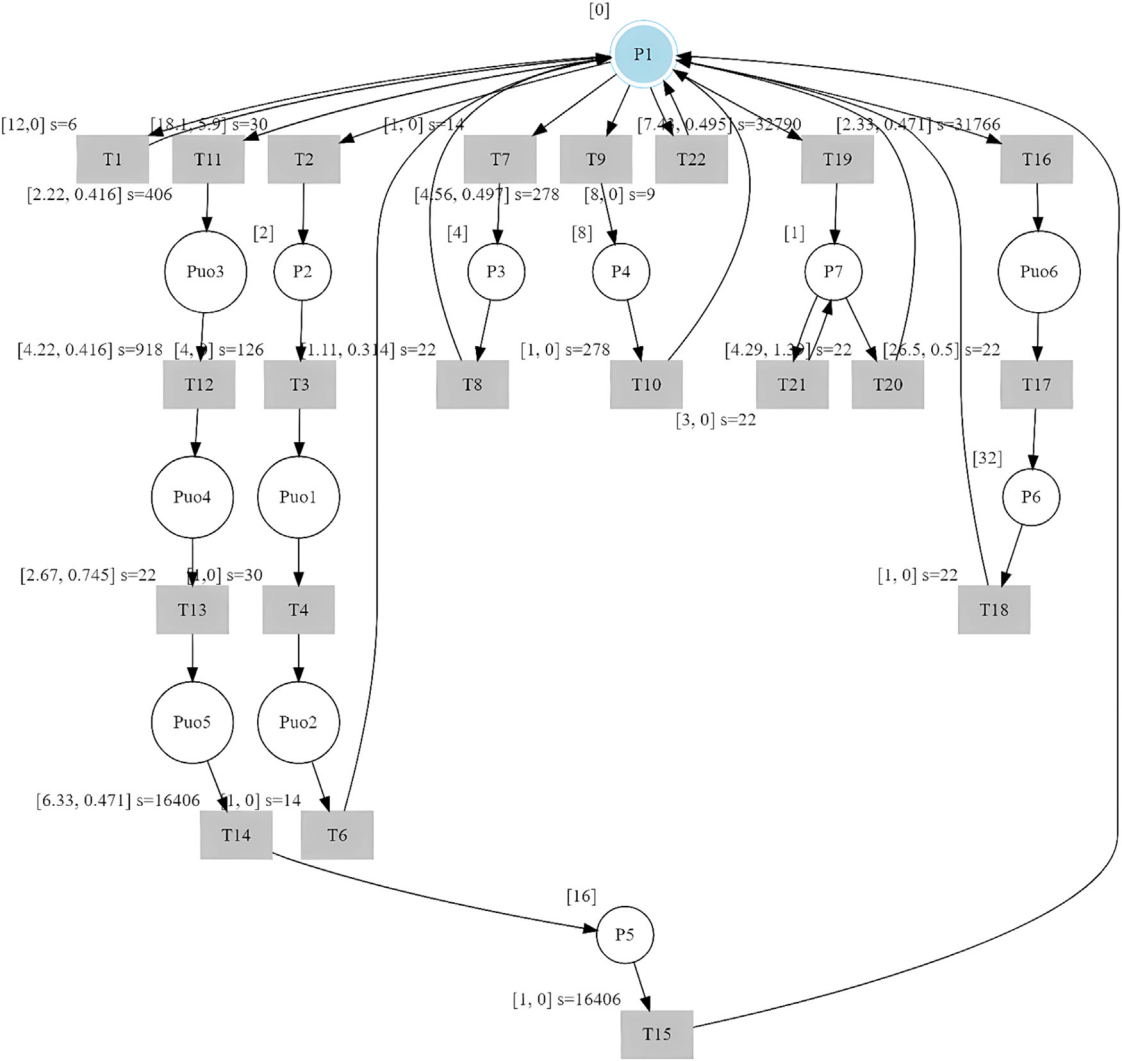
st-IPN of observable and unobservable behavior of the case study.

The st-IPN with observable and unobservable behavior is shown in Fig. 6.

### Discussion

By applying the method to identify observable behavior, the resulting model represents a dynamic system that reacts to the input signals, thus changing the outputs. Therefore, when there is no state change in the model before a sequence of input events related to the control commands, the proposed method infers the unobservable behavior by adding places to the observable model. In the identified PNs, which are shown in Figs. 4 and 6, with the inclusion of this type of places it was possible to eliminate sequences that did not belong to the system behavior, making $L_{o}=L_{g}$. Therefore, this model helps to represent system behaviors that could be closer to the real behavior of the system. Aspects are important when the resulting models are used to verify system performance, such as validating control strategies or fault diagnosis.

Conversely, this proposal allows for the modeling of causal and concurrent events. Causal events can be modeled because the observed sequences are generated in an orderly manner as the system is observed, and, in turn, the sequences are cyclic, and their representation in the st-IPN guarantees their order. Moreover, two events can be triggered simultaneously when more than one sequence is modeled. This situation can be observed in Fig. 6, when if place p6 is active, either transition $tr_{19}$ or $tr_{20}$ can be triggered. In the introductory example, another sequence of events can be used:

$$\left(\left(u_{34}y_{0}\right)\left(1,0\right)\right)\left(\left(u_{196}y_{0}\right)\left(8.3,0.2\right)\right)\left(\left(u_{192}y_{0}\right)\left(5.6,0.3\right)\right)\left(\left(u_{193}y_{1}\right)\left(9.2,0.25\right)\right)\left(\left(u_{34}y_{12}\right)\left(4.1,0,01\right)\right)\left(\left(u_{34}y_{0}\right)\left(9.95,0.2\right)\right).$$

**Fig. 7:**
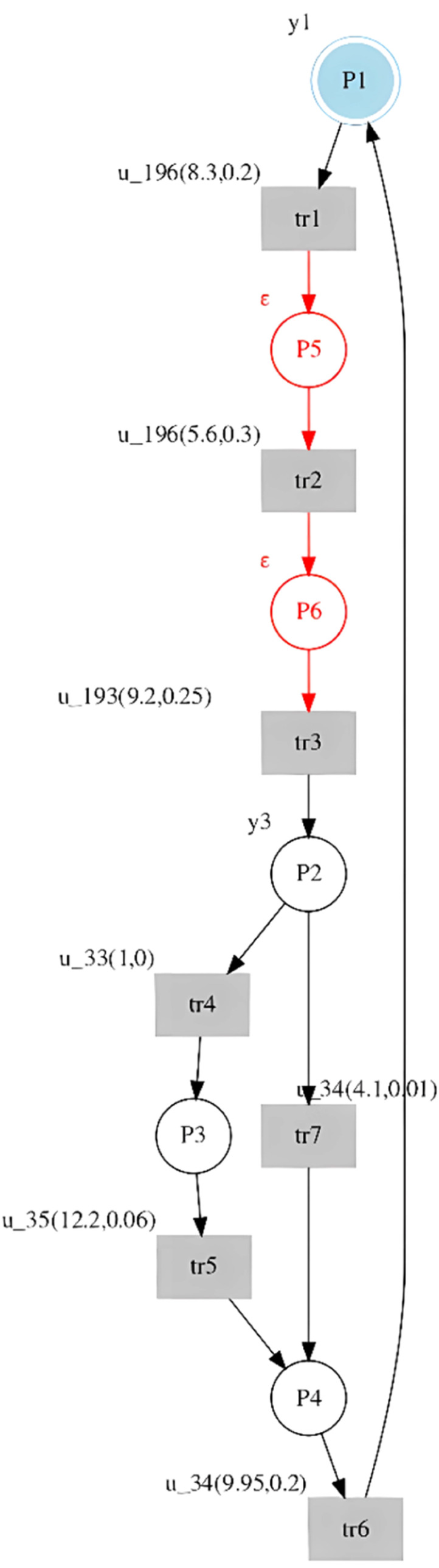
St-IPN with concurrent events.

The st-IPN for this sequence is shown in Fig. 7.With this variation, it is observed that when the place $p_{2}$ is active, the transitions $tr_{4}$ or $tr_{7}$ can be triggered; however only one can be triggered, and this depends on the controller's command.

## Conclusions

This paper presents an approach to solve the problem of inferring the unobservable behavior of a stochastic discrete event system. The method starts from the observation of the system and the generation of timed input-output events that represent the signals that are exchanged in a closed-loop DES. This method is applicable in DESs that react to the execution of input signals coming from the controller, with changes in the output signals. The method has two components: first, it identifies the structure of an st-IPN, the input and output functions associated with the PN and the temporal behavior of the transitions, and second, it infers the unobservable behavior based on language theory, namely by comparing the observed language with the one generated by the st-IPN and adding places for these languages to be the same.

In addition, the output function of the proposed method does not assign only the activation of one sensor reading to each place, but the combination of the activation of different readings. This condition is useful to improve the event-detectability of the resulting model; since to check this condition, the product of the incidence matrix by a matrix φ, which relates the sensor readings to the identified locations, must be found. Suppose the resulting matrix has linearly independent and non-zero columns, in that case, the system modeled in the st-IPN is event detectable, i.e., a unique identifier is associated with the triggering of each of the transitions.

### Limitations

The method has limitations, for example, it infers the observable and unobservable states in a DES, represented as an st-IPN; but not the unobservable transitions, on the other hand, the unobservable states are inferred from the reactive behavior of the input and output signals, when an input does not generate changes in the output; but it does not allow inferring if the unobservable behavior is due to some kind of failure, not necessarily due to lack of sensors.

### Future work

In future work, it is proposed to extend the method to detect and isolate faults in DES. Also a study comparing the resolution of the problem of inference of unobservable states in systems with low number of sensors, of the proposed method with other approaches such as neural networks.

## CRediT authorship contribution statement

**Rubén Santillán-Mosquera:** Investigation, Writing – review & editing, Conceptualization, Methodology. **Mariela Muñoz-Añasco:** Investigation, Writing – original draft, Validation, Conceptualization, Methodology.

## Declaration of Competing Interest

The authors declare that they have no known competing financial interests or personal relationships that could have appeared to influence the work reported in this paper.

## Data Availability

No data was used for the research described in the article.
